# Supplementary material for: Using a pacifier to decrease sudden infant death syndrome: an emergency department educational intervention
Source: PeerJ. 2014 Mar 13;2:e309. doi: 10.7717/peerj.309 (PMC3961164; doi:10.7717/peerj.309)
Supplement: Appendix S1 [file peerj-02-309-s001.pdf]

Initial Questionnaire

If other, specify

(4) Primary Caregiver? ☐ Mother ☐ Father ☐ Grandmother ☐ Other

(5) Age of primary caregiver? (if twoprimary caregiver use oldest)

(6) Race of primary caregiver? If other, specify

(7) Insurance type

(8) How many other children in home

(9) Number of bedrooms

(10) Baby's birth date (from other screen)

(11) Clinic Name If other, specify

(12) Did baby stay in NICU? ☐ yes ☐ no ☐ missing

(13) Did nurse/doctor discuss SIDS at discharge? ○ yes ○ no ○ doesn't remember ○ missing

(14) Has doctor discussed SIDS at checkups? ○ yes ○ no ○ doesn't remember ○ missing

(15) Which of the following were recommended:

- **(a)** Baby not sleep with adults? ○ yes ○ no ○ missing
- **(b)** Place baby on back? ○ yes ○ no ○ missing
- **(c)** No loose blankets? ○ yes ○ no ○ missing
- **(d)** No smoking? ○ yes ○ no ○ missing
- **(e)** Place pacifier in mouth to sleep? ○ yes ○ no ○ missing

(16) Given a pacifier by hospital or doctor? ○ yes ○ no ○ missing

(17) Does baby have pacifier? ○ yes ○ no ○ missing

(18) Does baby sleep with pacifier?

(19) If pacifier used, why? If other, specify

(20) If pacifier not used, why? If other, specify

(21) How is baby fed?

RA Entering data **RA who did the survey**

3 Month Follow Up Phone Survey

Due Date

Hello, my name is <your name> and I am a research assistant at Kern Medical Center.
I am trying to reach: .

If person is not reached:
Thank you for your time. We will try another day

If person is reached:
Hi I just had a few questions regarding your last visit to the er with (insert child's name) on . When you came one of our Research Assistants talked to you about Sudden Infant Death Syndrome. I just wanted to know (proceed to ask questions):

(1) How many people did you tell about pacifier use to prevent Sudden Infant Death Syndrome?

(2) Does your baby use a pacifier now?

(3) If a pacifier is not used during sleep, which of the following is the reason?
If other, please specify:

(4) Does your baby sleep on its back?

All 5 follow up phone calls failed? ☐ **yes** ☐ **no** ☐ **missing**

Follow up letter sent date:

Is 3 month follow up successful? ○ yes ○ no ○ missing

Follow up call 1 date		RA Name call 1	
Follow up call 2 date		RA Name call 2	
Follow up call 3 date		RA Name call 3	
Follow up call 4 date		RA Name call 4	
Follow up call 5 date		RA Name call 5	

Questionnaire | goto Demographics | Opening Screen

One year Follow Up Phone Survey

Due Date

Hello, my name is <your name> and I am a research assistant at Kern Medical Center.
I am trying to reach: .

If person is not reached:
Thank you for your time. We will try another day

If person is reached:
Hello, we are calling to thank you for filling in the survey about Sudden Infant Death Syndrome on . This call is to get an update on the information you received. Do you mind taking the time to answer a few more questions?

(1)How many ear infections has your child had?

(2)Did your baby haveany episoedes of turning blue or stopped breathing since we first told you about the study — ○ yes ○ no ○ m

(3) Did your baby die of SIDS ? — ○ yes ○ no ○ m

(4) Has your baby any other medical problems? — ○ yes ○ no ○ m

What are they?

(5) How is your child doing overall?

All 5 follow up phone calls failed? — ○ yes ○ no ○ missing

Follow up letter sent date:

Is one year follow up successful? — ○ yes ○ no ○ missing

Follow up call 1 date		RA Name call 1	
Follow up call 2 date		RA Name call 2	
Follow up call 3 date		RA Name call 3	
Follow up call 4 date		RA Name call 4	
Follow up call 5 date		RA Name call 5	

follow_up_questions_one_year ?

pacifier_f_call_q5_year
