# Supplementary material for: Using a pacifier to decrease sudden infant death syndrome: an emergency department educational intervention
Source: PeerJ. 2014 Mar 13;2:e309. doi: 10.7717/peerj.309 (PMC3961164; doi:10.7717/peerj.309)
Supplement: Appendix S2 [file peerj-02-309-s002.pdf]

- <u>Don't</u> force your infant to suck on the pacifier
- If you child is being breast fed, <u>wait</u> until he/she reaches one month of age before giving them a pacifier.

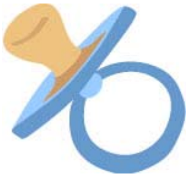

<u>Los Chupones y SIDS</u>

Estudios han demonstrado que el usode chupones para bebés 12 meses y menores disiminuido el riesgo de SIDS (**Síndrome de Muerte Súbita Infantil**). Se recomienda altamente que le dé un chupón a su bebé <u>antes de</u> dormir. Pero, no ponga el chupón en la boca de su bebé si ya está dormido son él.

<u>Cosas que se Deben de Observar</u>:

- Use un chupón limpio
- Cambie el chupón regularmente
- <u>No</u> le ponga nada dulce al chupón
- <u>No</u> forcé el chupón en la boca de su bebé
- Espera que su bebé cumpla un mes para usar el chupón si es que le esta dando pecho

Kern Medical Center's Emergency Department research staff tries to educate their patients and the public about ways to reduce Sudden Infant Death Syndrome (SIDS). Please help save the lives of our children by spreading the word about SIDS prevention. Thank you…

KERN MEDICAL CENTER EMERGENCY DEPARTMENT

El Centro Medico de Emergencia de el Conado de Kern esta tratando de educar a pacientes y al publico las maneras de cómo reducir el riesgo de Síndrome de Muerted Súbita Infantil (SIDS). Por favor ayúdenos a salvar la vida de nuestros nuestros niños pasando esta información a los demás. Muchas gracias…

<u>Sudden Infant Death Syndrome (SIDS) Prevention</u>

COUNTY OF KERN STATE OF CALIFORNIA

KERN MEDICAL CENTER EMERGENCY DEPARTMENT

Website/Pagina de Internet:
<u>www.kmcemed.edu</u>

What is Sudden Infant Death Syndrome?

Sudden Infant Death Syndrome (**SIDS**) occurs when an infant younger than one of age suddenly dies from an unknown cause.

¿Que es el Sídrome de Muerte Súbita Infantil?

El Síndrome de Muerte Súbita Infantil (**SIDS**), también conocido como la muerte de cuna, es cuando un infante de 12 meses de edad ó menos muere repentinamente de causa desconocida.

Sleeping Postitions & SIDS Prevention

One of the most important things you can do to help reduce the risk of SIDS is to lay your healthy baby on his or her back when putting him or her to sleep. Do this when your baby is being put down for a nap or to bed for the night. You may also turn your baby's head to either side.

The American Academy of Pediatrics recommends that babies sleep on their backs unless your doctor instructs you otherwise

Other ways to reduce the risk of SIDS include:

- Use a crib
- Don't use props, pillows, or anything else to hold baby in crib
- Avoid overdressing or overheating baby
- Avoid having your baby sleep with you if you are using drugs, alcohol, or cigarettes
- Avoid smoking around baby
- Don't use alcohol or drugs

* Be sure that anyone caring for you baby knows these guidelines.

Posición Para Dormir y Prevención del SIDS

Una de las cosas mas importantes que usted puede hacer para reducir el riesgo de SIDS es colocar a su bebé sano para dormir boca arriba. Cóloquelo de esta manera cada vez que ponga a su bebé a la cuna para dormir una siesta ó por la noche. Usted puede doblar la cabeza del bebé hacia uno de los lados.

La Academia Americana de Pediatría recomienda que los bebés duerman de esta manera, a no ser que su medico le haya dada otras intrucciones.

Otros modos de reducir el riesgo de SIDS son:

- Use una cuna
- No use almohadas, reclinadores, ó cualquier otra cosa para sujetar al bebé dentro de la cuna
- Evite vestir ó calendar desmasiado al bebé
- Evite que el bebé duerma con usted si usa drogas, alcohol, ó fuma
- No fume cerca de el bebé
- No use drogas ó alcohol

* Asegúrese que todas las personas que cuidan a su bebé sigan estas direcciones.

Best Sleep Position

Make sure your baby goes to sleep on his or her back, as this provides the **BEST** protection against SIDS.

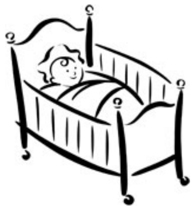

La Mejor Posición

Asegúrese que su bebé duerma boca arriba. Esto provee **la mejor** protección contra el SIDS.

Pacifiers & SIDS

Studies have shown that the use of pacifiers for babies 12 months and younger has decreased the rate of SIDS (**Sudden Infant Death Syndrome**). It his highly recommended that you give your infant a pacifier before a nap or sleep. Do not place the pacifier into your baby's mouth if they are already asleep without one.

Things to Watch Out For:

- Make sure to use a clean pacifier
- Replace pacifier on a regular basis
- Don't dip the pacifier into/onto anything sweet
