# Supplementary material for: Using a pacifier to decrease sudden infant death syndrome: an emergency department educational intervention
Source: PeerJ. 2014 Mar 13;2:e309. doi: 10.7717/peerj.309 (PMC3961164; doi:10.7717/peerj.309)
Supplement: Appendix S3 [file peerj-02-309-s003.docx]

**Appendix 3**

Univariate analysis of factors associated with primary caregiver knowledge of each of five pacifier prevention recommendations.

| ***Infant should sleep on his back*** |  |  |  |  |
| --- | --- | --- | --- | --- |
| **Factor** |  | **Did not know** | **Knew** | **p-value** |
| N |  | 167 | 613 |  |
| Male |  | 94 (56.3%) | 337 (55.0%) | 0.76 |
| Age, median (IQR) | months | 3.0 (0.8, 67) | 4.1 (1.3, 7.7) | 0.015 |
| Age of primary caregiver , median (IQR) | years | 25 (21, 31) | 25 (20, 31) | 0.33 |
| Primary Caregiver >35 years |  | 24 (14.4%) | 95 (15.5%) | 0.72 |
| Started pacifier in the hospital |  | 57 (34.1%) | 304 (49.9%) | <0.001 |
| SIDS counseling at discharge |  | 41 (26.8%) | 276 (48.4%) | <0.001 |
| SIDS counseling in the clinic |  | 27 (17.1%) | 204 (35.4%) | <0.001 |
| Number of bedrooms at home median (IQR) |  | 2 (2, 3) | 3 (2, 3) | 0.13 |
| Number of other children, median (IQR) |  | 1 (0, 3) | 2 (1, 3) | 0.63 |
| Pacifier user at enrollment |  | 63 (37.7%) | 268 (43.7%) | 0.16 |
| **Other recommendations known** |  |  |  |  |
| Not in bed with adult |  | 50 (29.9%) | 419 (68.5%) | <0.001 |
| No blankets, stuffed animals |  | 73 (43.7%) | 516 (84.7%) | <0.001 |
| No smoking |  | 110 (66.7%) | 550 (90.0%) | <0.001 |
| Pacifier when sleeping |  | 38 (22.8%) | 230 (37.7%) | <0.001 |

| ***Infant should not cosleep with an adult*** |  |  |  |  |
| --- | --- | --- | --- | --- |
| Factor |  | **Did not know** | **Knew** | **p-value** |
| N |  | 310 | 469 |  |
| Male |  | 173 (55.8%) | 257 (54.8%) | 0.78 |
| Age, median (IQR) | months | 3.4 (1.0, 7.2) | 4.2 (1.4, 7.85) | 0.075 |
| Age of primary caregiver , median (IQR) | years | 25 (21, 30) | 25 (21, 31) | 0.60 |
| Primary Caregiver >35 years |  | 39 (12.6%) | 80 (17.1%) | 0.089 |
| Started pacifier in the hospital |  | 118 (38.2%) | 242 (51.9%) | <0.001 |
| SIDS counseling at discharge |  | 88 (30.1%) | 228 (53.0%) | <0.001 |
| SIDS counseling in the clinic |  | 57 (19.3%) | 174 (39.6%) | <0.001 |
| Number of bedrooms at home median (IQR) |  | 3 (2, 3) | 3 (2, 3) | 0.059 |
| Number of other children, median (IQR) |  | 1 (1, 3) | 1 (1, 3) | 0.47 |
| Pacifier user at enrollment |  | 130 (41.9%) | 201 (42.9%) | 0.80 |
| **Other recommendations known** |  |  |  |  |
| Sleeps on back |  | 193 (62.3%) | 419 (89.3%) | <0.001 |
| No blankets, stuffed animals |  | 183 (59.2%) | 405 (86.9%) | <0.001 |
| No smoking |  | 237 (77.2%) | 422 (90.2%) | <0.001 |
| Pacifier when sleeping |  | 78 (25.2%) | 189 (40.5%) | <0.001 |

| ***No blankets/stuffed toys in crib*** |  |  |  |  |
| --- | --- | --- | --- | --- |
| **Factor** |  | **Did not know** | **Knew** | **p-value** |
| N |  | 187 | 589 |  |
| Male |  | 98 (52.4%) | 332 (56.4%) | 0.34 |
| Age , median (IQR) | months | 3.6(1.0, 7.1) | 4.0 (1.3, 7.6) | 0.35 |
| Age of primary caregiver, median (IQR) | years | 25 (21, 31) | 25 (21, 31) | 0.87 |
| Primary Caregiver >35 years |  | 30 (16.0%) | 89 (15.1%) | 0.76 |
| Started pacifier in the hospital |  | 63 (33.9%) | 296 (50.5%) | <0.001 |
| SIDS counseling at discharge |  | 46 (26.4%) | 270 (49.5%) | <0.001 |
| SIDS counseling in the clinic |  | 39 (22.3%) | 191 (34.4%) | 0.003 |
| Number of bedrooms at home median (IQR) |  | 3 (2, 3) | 3 (2, 3) | 0.97 |
| Number of other children, median (IQR) |  | 1 (0, 3) | 2 (1, 3) | 0.72 |
| Pacifier user at enrollment |  | 64 (34.2%) | 266 (45.2%) | 0.008 |
| **Other recommendations known** |  |  |  |  |
| Not in bed with adult |  | 61 (32.6%) | 405 (68.9%) | <0.001 |
| Sleeps on back |  | 93 (49.7%) | 516 (87.6%) | <0.001 |
| No smoking |  | 99 (53.2%) | 557 (95.1%) | <0.001 |
| Pacifier when sleeping |  | 38 (20.3%) | 230 (39.2%) | <0.001 |

| ***Caregivers should not smoke*** |  |  |  |  |
| --- | --- | --- | --- | --- |
| **Factor** |  | **Did not know** | **Knew** | **p-value** |
| N |  | 116 | 660 |  |
| Male |  | 68 (58.6%) | 361 (54.7%) | 0.43 |
| Age, median (IQR) | months | 3.4(1.0, 6.7) | 4.0 (1.3, 7.6) | 0.23 |
| Age of primary caregiver, median (IQR) | years | 26 (21, 33) | 25 (20, 31) | 0.064 |
| Primary Caregiver >35 years |  | 23 (19.8%) | 96 (14.5%) | 0.15 |
| Started pacifier in the hospital |  | 47 (40.9%) | 311 (47.3%) | 0.20 |
| SIDS counseling at discharge |  | 28 (26.4%) | 289 (47.1%) | <0.001 |
| SIDS counseling in the clinic |  | 24 (22.0%) | 207 (33.3%) | 0.020 |
| Number of bedrooms at home median(IQR) |  | 3 (2, 3) | 3 (2, 3) | 0.38 |
| Number of other children, median (IQR) |  | 2 (1, 3) | 1 (0, 3) | 0.21 |
| Pacifier user at enrollment |  | 45 (38.8%) | 282 (42.7%) | 0.43 |
|  |  |  |  |  |
| **Other recommendations known** |  |  |  |  |
| Not in bed with adult |  | 46 (39.7%) | 422 (64.0%) | <0.001 |
| Sleeps on back |  | 61 (52.6%) | 550 (83.3%) | <0.001 |
| No smoking |  | 0 (0.0%) | 660 (100.0%) | <0.001 |
| Pacifier when sleeping |  | 19 (16.4%) | 246 (37.4%) | <0.001 |

| ***Offer a pacifier when putting to sleep*** |  |  |  |  |
| --- | --- | --- | --- | --- |
| **Factor** |  | **Did not know** | **Knew** | **p-value** |
| N |  | 509 | 268 |  |
| Male |  | 272 (53.4%) | 156 (58.2%) | 0.20 |
| Age, median (IQR) | month | 3.4(1.0, 7.0) | 5.0(1.6, 8.3) | 0.001 |
| Age of primary caregiver, median (IQR) | years | 25 (21, 30) | 26 (21, 31) | 0.19 |
| Primary Caregiver >35 years |  | 74 (14.5%) | 45 (16.8%) | 0.41 |
| Started pacifier in the hospital |  | 210 (41.5%) | 151 (56.3%) | <0.001 |
| SIDS counseling at discharge |  | 183 (38.4%) | 133 (54.7%) | <0.001 |
| SIDS counseling in the clinic |  | 121 (25.2%) | 109 (43.3%) | <0.001 |
| Number of bedrooms at home median(IQR) |  | 3 (2, 3) | 2 (2, 3) | 0.17 |
| Number of other children, median (IQR) |  | 1 (1, 3) | 2 (1, 3) | 0.70 |
| Pacifier user at enrollment |  | 204 (40.1%) | 125 (46.6%) | 0.078 |
| **Other recommendations known** |  |  |  |  |
| Not in bed with adult |  | 278 (54.6%) | 189 (70.8%) | <0.001 |
| Sleeps on back |  | 380 (74.7%) | 230 (85.8%) | <0.001 |
| No smoking |  | 411 (80.9%) | 246 (92.8%) | <0.001 |
| No blankets, stuffed animals |  | 356 (70.5%) | 230 (85.8%) | <0.001 |
