# Supplementary material for: Using a pacifier to decrease sudden infant death syndrome: an emergency department educational intervention
Source: PeerJ. 2014 Mar 13;2:e309. doi: 10.7717/peerj.309 (PMC3961164; doi:10.7717/peerj.309)
Supplement: Appendix S4 [file peerj-02-309-s004.docx]

Appendix 4. Multivariate analysis of the factors from Appendix 3

Factors with p value <0.2 in univariate analysis were entered into logistic regression models. Ultimately only items with a p value <0.5 and models with satisfactory goodness of fit, acceptable variance inflation factors, and without collinearity were retained.
